# Reusing data from HL7 CDA-based shared EHR systems for clinical trial conduct: a method for analyzing feasibility

**DOI:** 10.1186/s12911-025-02980-2

**Published:** 2025-04-01

**Authors:** Georg Duftschmid, Florian Katsch, Gabriela Ciortuz, Dipak Kalra, Christoph Rinner

**Affiliations:** 1 Center for Medical Data Science (CEDAS), Medical University of Vienna, Spitalgasse 23, Vienna, 1090 Austria; 2 Ludwig Boltzmann Institute for Digital Health and Prevention, Salzburg, Austria; 3 Institute of Medical Informatics, University of Luebeck, Lübeck, Germany; 4 European Institute for Innovation through Health Data (i~HD), University College of London, London, UK; 5 Visiting Professor at University of Gent, Gent, Belgium; 6 Austrian Agency for Health and Food Safety GmbH (AGES), Vienna, Austria

**Keywords:** Electronic medical records, Clinical trials, Data models, Data collection

## Abstract

**Background:**

Electronic health record (EHR) systems have been shown to represent a valuable source of data reuse in the design and conduct of clinical trials. Earlier work has mostly focused on institutional EHR systems. Shared EHR systems have been neglected so far, even though they are highly prevalent today and their characteristics (integrated data across a patient’s care providers, standardized information model) make them attractive for the task. However, as they typically focus on a limited data set for the most common care situations, it remains unclear, whether shared EHR systems actually cover the data elements required for clinical trial conduct. In this paper we present a method, which allows shared EHR systems to be analyzed in this regard.

**Methods:**

We focus on shared EHR systems using HL7 CDA as this is currently the most-widely used content standard. For the data elements that are commonly used in clinical trials we refer to the EHR4CR reference list. The latter is semiautomatically mapped to the EHR system’s information model using the open source tool ART-DECOR. For the final automatic analysis of the mappings, another open source tool is provided.

**Results:**

A stepwise approach was developed to analyze HL7 CDA-based shared EHR systems for their coverage of data elements that are relevant for clinical trials. All tools used in this work as well as all mappings are publicly accessible to make the method reusable and the results reproducible. We applied our approach to the Austrian nation-wide EHR system ELGA and showed that the latter allows the recording of 88% of all EHR4CR data elements, 77% in structured format.

**Conclusions:**

Our method allows HL7 CDA-based shared EHR systems to be easily analyzed to what extent their content could be reused in the context of clinical trials. The results for ELGA indicate that it has a substantial corresponding potential.

**Clinical trial number:**

Not applicable.

## Background

Electronic health record (EHR) systems are a valuable source of routine data that have the potential to be reused in clinical research [1]. A particularly rewarding application area is clinical trials, where routine data can be leveraged for the different phases of trial design and conduct [2]. Within the trial process phase, feasibility checking [3], patient recruitment [4], and trial execution [5] can benefit from the reuse of routine data.

Data reused for trial conduct typically originates from institutional EHR systems, such as hospital information systems [6] or systems from outpatient care providers [7]. As institutional EHR systems typically store their data in proprietary formats, automatic processing of these data is frequently implemented in a site-specific way and can therefore not be directly applied to data recorded at other institutions. This is, however, a substantial limitation amongst others in the context of multicenter clinical trials, where several institutions cooperate in the conduct of a common trial. A common solution to this problem is to employ a common data model to which all proprietary data of the cooperating institutions are mapped [8].

Within the EHR4CR project, a common data model and a suite of IT tools were developed that support an inter-institutional reuse of EHR data for clinical trials [9]. Further common data models [10–12] are applied in the context of EHR-based phenotyping, which has the task of cohort identification in common with clinical trial conduct. The Observational Medical Outcomes Partnerships (OMOP) common data model has been used by several researchers as the target representation of an automatic transformation process of free text trial eligibility criteria into a computer-readable format [13, 14]. Even though the community of institutions employing common data models within research networks is growing, the number is still limited due to the substantial up-front effort required for transforming the institutional data into a common data model.

Shared EHR systems integrate health information from different care providers of a patient [15]. As they aim for an inter-institutional integration of health information, they typically employ a standardized information model. This standardized information model represents an immediate advantage when it comes to reusing the data for trial conduct. It can serve as a common data model, i.e. algorithms processing the underlying data can be shared between all participants of the shared EHR system. In contrast to a research network, however, the task of transforming an institution’s internal data to the common data model was already concluded when joining the shared EHR system, no additional transformation efforts are required.

If the shared EHR system achieves a wide geographical coverage and high participation rate of care providers, another advantage takes effect. The shared EHR system can then provide a nearly complete view on those data elements of a patient’s medical history, which are recorded within the system. In contrast, a research network will typically include a limited set of health institutions – these may cover detailed information that were recorded within the institutions but may also lack a substantial part of routine data on their patients that were recorded by care providers not participating in the network. A nation-wide EHR system with mandatory participation of care providers would thus represent an ideal scenario for our use case. According to a recent survey, 87% of the European WHO member states employed a nation-wide EHR system in 2023 [16]. The Bertelsmann Stiftung provides another interesting source for an international comparison of digital health implementations that also considers EHR systems [17].

Shared EHR systems, however, typically focus on collecting a limited set of data elements that is expected to deliver the most relevant data in regular care situations. It therefore remains unclear, whether shared EHR systems actually cover the data elements required for clinical trial conduct and can thus gain the before-mentioned advantages.

In this paper we present an approach, which allows shared EHR systems to be analyzed in this regard. We focus on EHR systems using the HL7 Clinical Document Architecture (CDA) [18] as this is currently the most-widely used content standard [19]. In order to ensure easy reuse of our approach, it is exclusively based on open source tools. We demonstrate the application of our methodology by analyzing Austria’s nation-wide EHR system ELGA [20].

ELGA has been operational since 2015. All public care providers (hospitals, outpatient care providers, and pharmacies) are obliged to participate in the system. With respect to participation of citizens, ELGA pursues an opt-out approach, i.e. each citizen participates in ELGA per default, unless an opt-out is actively enacted. Currently, around 97% of the Austrian population participate in ELGA. The HL7 CDA standard forms the basis of ELGA’s information model. In an earlier work we examined whether ELGA data can be utilized for patient recruitment [21]. In the present paper, we focus on making the underlying methodology available for reuse for other shared EHR systems and further extend ELGA’s analysis by also considering the use cases of trial feasibility checking and trial execution.

## Methods

Our goal is to systematically analyze whether a given shared EHR system contains the data that are relevant for clinical trial conduct. In order to answer this question, we perform the following steps:

1. Identify the data elements that are relevant in the context of clinical trials.
2. Consolidate these data elements in a formal concept repository that forms the source of the following mapping.
3. Map concept repository to EHR system’s information model.
  - Automatically identify components of the EHR system’s information model that represent suitable mapping targets based on associated semantic annotations.
  - Manually map source concepts for which EHR system target components lack semantic annotations.
4. Automatically analyze mappings for an overview of EHR system’s coverage of data elements that are relevant in the context of clinical trials.

In the following we will explain the implementation of each of these steps in detail.

### Identification of data elements that are relevant for clinical trial conduct

The European EHR4CR project [9], which was completed in 2016, aimed to support clinical trial conduct by reusing routine data from EHRs. The project has identified data elements that are commonly needed for trial feasibility checking [22], patient identification / recruitment [23], and trial execution [24]. Within the medical data models (MDM) portal [25], the data elements for the three use cases can be viewed [26–28]. The data set for trial feasibility checking contains 75 data elements. Those for patient identification / recruitment and for trial execution contain 149 and 133 items, respectively. Each data element is associated with a data type. Semantics are clarified by free text descriptions as well as UMLS codes. Within the present paper, we refer to these three datasets as our reference lists of data elements that are relevant for clinical trial conduct.

### Creation of a web-based concept repository for clinical trial conduct

As a preparatory step for analyzing EHR systems’ coverage of the three reference lists, we created an ART-DECOR concept repository for all contained data elements. ART-DECOR [29] is an open source tool that supports various tasks in the context of health information exchange. One of these tasks is the specification of the underlying data elements in the form of so-called concepts. Similar to MDM, concepts are associated with a data type and a free text description. Concept semantics can additionally be expressed by means of codes from standardized terminologies. Concepts may further be hierarchically grouped and inherited in other ART-DECOR projects.

This inheritance mechanism is a key strength of ART-DECOR, which allows our concept repository to be reused in the analysis of any particular EHR system for its utility in clinical trial conduct. Another reason for applying ART-DECOR was that it supports the management of various HL7 CDA artefacts.

When building our concept repository, we reproduced the data elements of the three EHR4CR reference lists according to the information provided in the corresponding MDM projects. Textual descriptions are only partially available in the MDM projects and were thus taken from [24] as far as possible. UMLS codes are available in the MDM projects for 308 (86%) of a total of 357 data elements. For some of the data elements, SNOMED CT codes are provided in [24]. We associated all UMLS and SNOMED CT codes with our ART-DECOR concepts.

As ART-DECOR covers a broader spectrum of data types than MDM, we selected more specific data types where appropriate:

- Quantities expressed with MDM data types Float, Integer, or Text (e.g., for lab values or vital signs) were mapped to data type Quantity in ART-DECOR.
- Codes expressed with MDM data types String, Text, or Integer (e.g., for gender or procedure codes) were mapped to data type Code in ART-DECOR.
- Counts expressed with MDM data types Integer or Float (e.g., for “number of pregnancies”, “years smoked”) were mapped to data type Count in ART-DECOR.
- In a few cases, seemingly erroneous MDM data types were changed in ART-DECOR (e.g., “diagnosis code” was changed from Date to Code, “currently breast feeding” was changed from Text to Boolean, “date of assessment” was changed from Text to Date).

Our concept repository is called “EHR4CR Data Inventory” and is publicly accessible [30]. It includes individual datasets that hold the concepts for the three use cases (i) feasibility checking, (ii) patient identification and recruitment, and (iii) trial execution. The three EHR4CR reference lists share several data elements. We therefore created a fourth dataset named “EHR4CR Basic Data Elements” in our concept repository, which holds all shared data elements. These data elements are then included in the other three datasets via inheritance where appropriate.

### Expressing an EHR system’s coverage of concept repository

As mentioned before, we focus on EHR systems based on the HL7 CDA standard. This means that the data is organized in CDA documents of different types. The content and structure of CDA document types are specified by means of the HL7 templates standard [31]. We formally express the EHR system’s coverage of our trial-related concept repository by mapping our concepts to those elements of the EHR system’s CDA templates, which hold the corresponding data. This mapping is performed by means of the ART-DECOR “template association” function.

### Mapping the EHR4CR reference lists to the EHR system’s CDA templates

When searching for semantically equivalent CDA template elements for the data elements of the EHR4CR reference lists, we first apply an automatic code-based matching procedure. Hereby, the goal is to identify CDA templates associated with a code that is equivalent to one of the codes associated with the concepts of our repository. This would suggest that the template is semantically comparable to the concept and can thus be expected to hold the required data.

Our automatic matching procedure leverages the extensive mappings between standardized code systems that have been implemented in the OHDSI community [32]. In [33], the UMLS vocabulary was mapped to standard OMOP concepts. Our automatic matching procedure expands the UMLS codes from our concept repository based on [33] and the predefined mappings to the different code systems that exist in the OMOP vocabulary for standard concepts. It then scans the CDA templates of the examined EHR system for matches with one of the codes of the expanded list. The Python script of the automatic matching is publicly available in our “procedure repository” [34].

The automatic matching will only deliver a partial mapping of our concept repository, if not all of the EHR system’s templates are associated with codes. The remaining data elements of our concept repository will then have to be mapped manually. Implementers of this task have to be familiar with the CDA templates of the examined EHR system.

### Analysis of the mappings

**Fig. 1:**
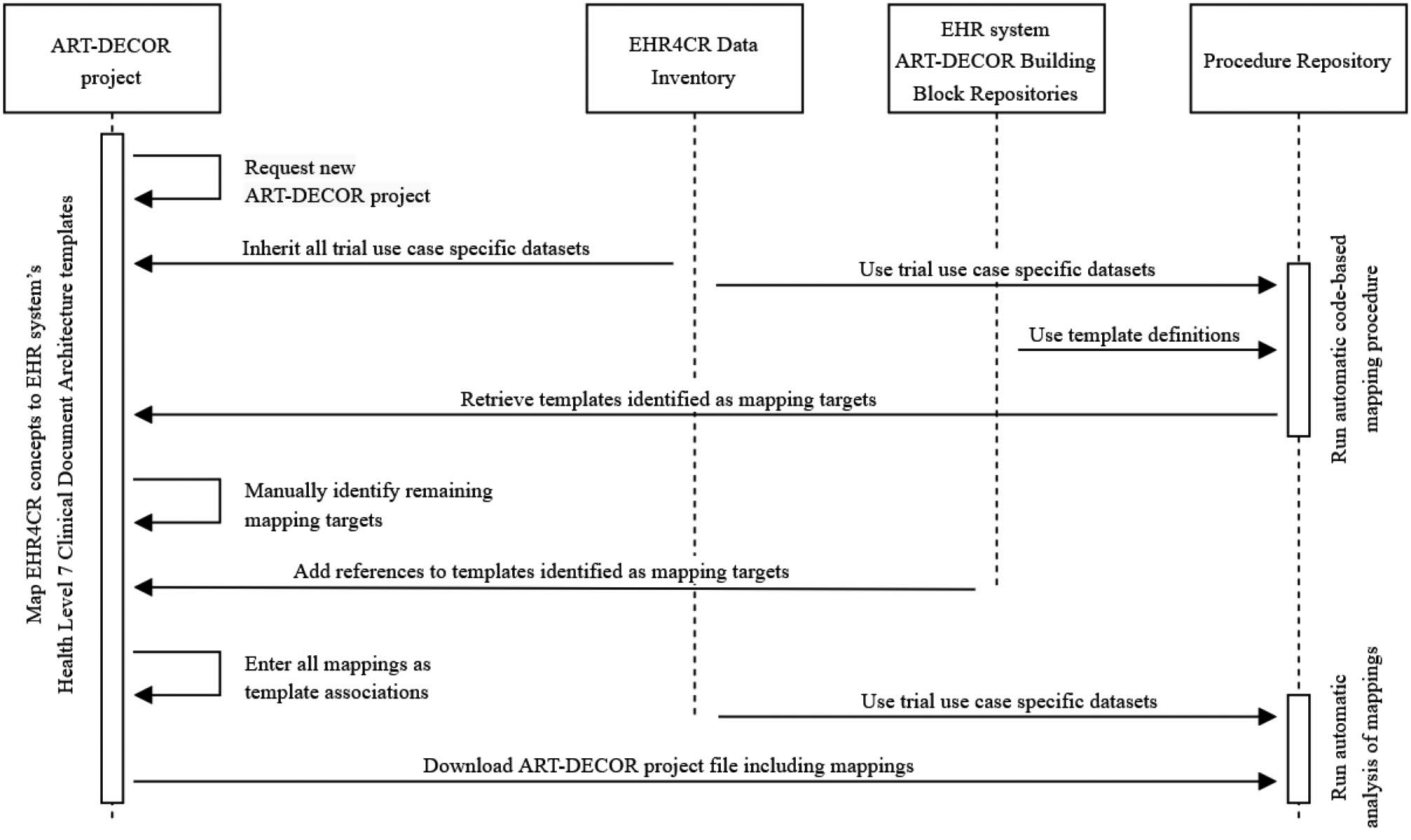
Workflow of analyzing an EHR system for coverage of trial-specific concepts

All mappings are formally stored as template associations within an ART-DECOR project. The complete project file including the template associations can be downloaded in XML format via the ART-DECOR REST API. For an automated analysis of the mappings, we implemented a Python script. It reads the ART-DECOR project file “EHR4CR Data Inventory” as well as the ART-DECOR project file containing the mappings and generates a detailed analysis of the EHR system’s coverage of the EHR4CR reference lists in YAML format. The Python script is publicly available in our “procedure repository” [34]. Figure 1 depicts an overview of the complete workflow of our method.

The analysis focuses on the number and percentage of concepts that could be mapped to at least one template element. Hereby it distinguishes mapping targets containing structured versus free text content. If a concept is mapped to a structured and a free text template element simultaneously, the mapping is considered as structured. The analysis is done on the level of the three use cases (feasibility checking, patient identification and recruitment, trial execution) as well as on the level of the concept groups within the use cases.

## Results

In the following we demonstrate the application of our methodology by analyzing Austria’s nation-wide EHR system ELGA.

### Expressing ELGA’s coverage of concept repository

**Fig. 2:**
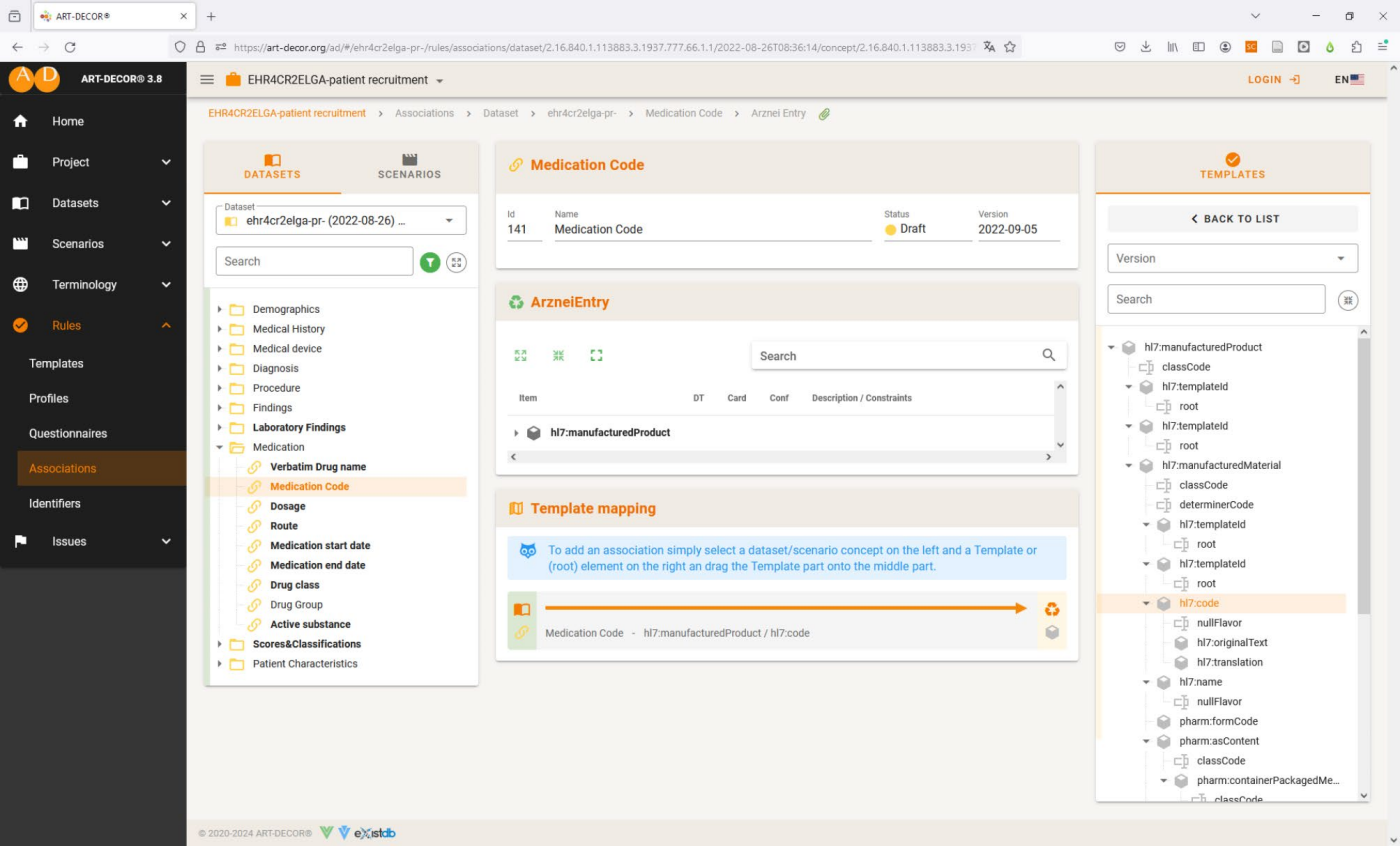
Example of mapping concept “Medication Code” to element “hl7:code” of template “Arznei Entry” as a template association

We created three ART-DECOR projects, which separately hold the mappings for the use cases feasibility checking [35], patient identification and recruitment [36], and trial execution [37]. In each project we inherited all concepts of the corresponding use case from the “EHR4CR Data Inventory”. We further referenced all ELGA templates, which represent targets of our mappings. We only used ELGA templates that are referred to in ELGA document types of status “Normative”. This ensures that the templates are actually applied in CDA documents that are currently part of ELGA. All ELGA templates used in our work are publicly available within three ART-DECOR “Building Block Repositories (BBR)” [38–40]. The mappings are stored in a structured way in the ART-DECOR project file (see Fig. 2), from where they can be retrieved for further processing via a REST API.

### Mapping the EHR4CR reference lists to ELGA templates

In the course of our automatic code-based mapping procedure we found matching codes within the three ELGA BBRs for 85 codes associated with concepts of our repository. The matches were manually validated by removing duplicates due to multiple versions of the same value set, grouping codes that belong to the same value set, and removing matches where manual verification showed that the identified ELGA template was not semantically equivalent to the corresponding concept of our repository. Based on the remaining matches, we were able to identify mappings for 75 concepts. The automatic matching procedure thus covered the mapping of 21% of the 357 concepts within our three use cases (i) feasibility checking, (ii) patient identification and recruitment, and (iii) trial execution. The remaining concepts were then manually mapped separately by GD, CR, and GC, compared and harmonized.

Frequently, more than one template was found to be a potential data source for one of our concepts. In this case, multiple template associations were stored to represent the corresponding mappings. As an example, the concept “Alcohol Abuse” could be recorded in free text in ELGA template “Lifestyle - uncoded” as well as in the form of an ICD10 diagnosis code for alcohol related disorders in ELGA template “Problem Entry”. Multiple template associations were also needed to represent a single mapping for “generic templates”, i.e. templates that can represent different concepts depending on the assignment of their element “code”. As an example, template “Laboratory Observation” is a generic template that can be customized to represent any lab measurement by setting its element “code” to the particular LOINC code of the lab parameter. The lab value is stored in the template’s element “value”. For instance, when looking for “Albumin” measurements in ELGA CDAs, we have to query the element “value” of “Laboratory Observation” instances, whose element “code” is set to LOINC code “1751-7 (Albumin)”. All concepts representing lab measurements were thus mapped to elements “code” and “value” of template “Laboratory Observation”.

### Analysis of the mappings

Table 1 depicts the analysis of the mappings for all concepts for the three use cases feasibility checking, patient identification and recruitment, and trial execution as well as an overall analysis. We validated the results of our automatic analysis based on spot checks.

**Table 1:** Portions of the concepts of the 3 use cases that could be mapped to ELGA template elements (total mappings versus mappings to structured elements)

	Concepts in use case	Concepts mapped	Concepts mapped to structured elements
Feasibility checking	75	71 (95%)	67 (89%)
Patient identif./recruitment	149	135 (91%)	119 (80%)
Trial execution	133	109 (82%)	89 (67%)
Overall	357	315 (88%)	275 (77%)

Table 2 shows the analysis of the mappings for the three use cases on the level of concept groups.

**Table 2:** Portions of the concepts per group within the three use cases that could be mapped to ELGA template elements (total mappings versus mappings to structured elements)

	Concepts in group	Concepts mapped	Concepts mapped to structured elements
**Feasibility checking**			
Demographics	5	5 (100%)	5 (100%)
Medical history	8	7 (88%)	4 (50%)
Diagnosis	4	3 (75%)	3 (75%)
Procedure	3	1 (33%)	0 (0%)
Findings	7	7 (100%)	7 (100%)
Laboratory findings	41	41 (100%)	41 (100%)
Medication	7	7 (100%)	7 (100%)
**Patient identif. / recruitment**			
Demographics	5	5 (100%)	5 (100%)
Medical history	10	7 (70%)	4 (40%)
Medical device	1	1 (100%)	0 (0%)
Diagnosis	5	4 (80%)	4 (80%)
Procedure	3	1 (33%)	0 (0%)
Findings	25	18 (72%)	16 (64%)
Laboratory findings	81	81 (100%)	81 (100%)
Medication	9	8 (89%)	8 (89%)
Scores & classification	9	9 (100%)	0 (0%)
Patient characteristics	1	1 (100%)	1 (100%)
**Trial execution**			
Demographics	4	2 (50%)	2 (50%)
ECG	9	0 (0%)	0 (0%)
Adverse events	11	5 (45%)	5 (45%)
Medical history	4	4 (100%)	1 (25%)
Disease characteristics	2	2 (100%)	2 (100%)
Disposition	2	0 (0%)	0 (0%)
Patient reported outcome	3	2 (67%)	0 (0%)
Vital signs	8	8 (100%)	7 (88%)
Laboratory	57	57 (100%)	57 (100%)
Lab data	6	6 (100%)	4 (67%)
Concomitant medication	9	8 (89%)	8 (89%)
Surgery	5	4 (80%)	0 (0%)
Substance use	8	8 (100%)	0 (0%)
Tumor resp.	5	3 (60%)	3 (60%)

## Discussion

According to Table 1, ELGA allows the recording of close to 90% of all EHR4CR data elements and around three quarters of all EHR4CR data elements can be stored in ELGA in structured form. From the three use cases, data elements that are relevant for “feasibility checking” achieve the highest coverage, indicating that ELGA could unfold its potential most effectively in this first phase of clinical trial conduct.

The main reason for ELGA’s good coverage of the EHR4CR data elements is that ELGA supports the structured recording of laboratory measurements and medication data, which account for 50% and 7% of the EHR4CR data elements. Further groups of EHR4CR data elements for which ELGA allows the recording of structured data to a high extent are demographics, findings, diagnoses, and vital signs. Groups, where data can be expected to be found primarily in free text format, are scores & classification, patient reported outcomes, surgery, substance use, and medical history. Groups, where ELGA currently provides minimal or no data coverage, are ECG, procedure, disposition, and adverse events. Accordingly, there are some obvious starting points for future extensions of ELGA with respect to its secondary use in the context of clinical trials. Independently of ELGA, a general lesson learned is that, due to their broad representation in the EHR4CR reference lists, a good coverage of laboratory parameters, medication data, and findings seems to be a key prerequisite for an EHR system’s ability to be reused in clinical trial conduct.

Our method is limited insofar, as it just describes to what extent an EHR system theoretically allows data elements to be recorded that are relevant in the context of clinical trial conduct. Our method does not consider actual data availability, i.e. which of the examined data elements are actually filled in by clinicians in clinical documentation practice. It can serve as a first check, whether the EHR system is prepared to deliver data for clinical trials without requiring access to the actual data and the rigorous measures to ensure their privacy.

In 2016, Ateya and colleagues rated 2,619 data elements from 228 primary care clinical trials for the availability of corresponding source data within EHR systems of general practitioners [7]. They estimated that 74% of the data elements, which were used in the context of patient identification and recruitment, would be likely available in structured format. In contrast to the present study, their results are based on expert opinion, a formal mapping between the clinical trial data elements and the EHR systems is not provided.

In 2013, Köpcke and colleagues compared 706 data elements from eligibility criteria used in 15 clinical trials with the data catalogs of 5 university hospital EHR systems [6]. They found that the EHR systems allow the documentation of 55% of the data elements. When analyzing data completeness, they reported that only 35% of the data elements were actually populated within the EHR systems. In contrast to [6], the present study analyzes a nation-wide shared EHR system for its coverage of trial-specific concepts.

In 2021, Melzer and colleagues analyzed the data warehouse of a university hospital for the existence of 70 data elements referenced in the eligibility criteria of a clinical trial and found a coverage of 75.7% [41]. They also checked data completeness and found that for their test cohort of 106 patients, only 26.9% of the data elements were actually recorded within the data warehouse. Even though the coverage rate found in [41] is similar to ours, their analysis focused on a single institutional EHR system, whereas the present study examines a nation-wide shared EHR system.

In 2011, El Fadly and colleagues analyzed a hospital EHR system for the availability of 232 data elements processed in the course of a multi-center clinical trial and found a coverage of only 13.4% [5]. They explain their low percentage with the automatic mapping procedure applied by them, which missed several actual matches, e.g. due to different labeling of semantically equivalent data elements. Again, the scope of El Fadly and colleagues’ analysis (single hospital EHR system) differs from our study (nation-wide shared EHR system).

In 2018, Butler and colleagues analyzed to what extent EHR data contained in OHDSI’s Synthesized Public Use File (SynPUF) cover 4,260 data elements that are referred to within eligibility criteria of 1,587 Alzheimer disease clinical trials [42]. They found 60% of the eligibility criteria data elements to be present in the SynPUF. The 4,260 data elements were automatically associated with SNOMED CT terms using a natural language processing application. Butler and colleagues focused on the analysis of synthetic EHR data, whereas the present study focuses on the analysis of the prescribed data structures of an existing nation-wide shared EHR system.

In 2019, we presented our preliminary results of ELGA’s coverage of the EHR4CR data elements for patient identification and recruitment [21]. Since then, availability of the data elements in structured format has increased from 61 to 79%, respectively from 5 to 12% in free text format. This is due to 3 additional document types from the domains ambulatory care, vaccination, and telemonitoring that have since been integrated into ELGA.

Even though HL7 CDA is still one of the most used standards in the domain of EHR data exchange [19], HL7 FHIR [43] has gained increasing significance within the last years and can be expected to eventually replace HL7 CDA within the next years. FHIR features a similar technology to CDA templates for specifying, which content of the predefined standardized components shall actually be used within a particular use case. These so-called FHIR profiles will be supported in the upcoming version 3.9 of ART-DECOR. It will then also be possible to map the data elements of our EHR4CR Data Inventory to FHIR profiles. Our Python-based scripts will of course have to be adapted to process FHIR profiles instead of CDA templates within ART-DECOR.

We have argued that a shared EHR system’s standardized data model, more precisely the HL7 CDA templates prescribed by the system, could serve as the common data model for trial-related tasks. Algorithms for trial-specific processing could be implemented that directly reference the template-based CDA data structures and could then be applied by all participants of the shared EHR system. An alternative could be to transform the EHR system’s CDA data model to a widespread common data model and then be able to apply already existing tools and algorithms for the common data model. As the transformation would only have to be implemented once and could then be reused by all shared EHR system participants, the corresponding effort seems acceptable. We have shown a corresponding transformation from the ELGA CDA templates to the OMOP common data model [44, 45].

## Conclusions

We presented a method that allows HL7 CDA-based shared EHR systems to be analyzed to what extent their content could be reused in the context of clinical trials. It exclusively applies open source tools and the generated results are fully reproducible:

(i) The mapping of clinical trial data elements to the EHR system’s information model is supported by an automatic code-based matching procedure. Mappings are formally represented as template associations in a publicly accessible ART-DECOR project. Reported coverage numbers of the EHR system are automatically derived from these mappings, no hidden manual processing is involved. The mappings may also be used in future work to derive XPaths for the retrieval of data from the EHR system to automatically check a trial’s inclusion/exclusion criteria.
(ii) Our analysis is based on a published reference list of data elements found to be relevant in a wide variety of trials. The results should thus have a broader explanatory power than referring to data elements of a small number of arbitrarily selected trials.
(iii) All tools used in this work are publicly available at [34]. In this repository we describe in detail the sequence of steps required to analyze another EHR system.

Based on our results for ELGA we conclude that it has the theoretical potential to provide a substantial contribution for the conduct of clinical trials. In order to get some insight in the practical documentation of EHR4CR data elements in ELGA, we aim in the next step to examine available ELGA data of patients, who were manually checked for participation in clinical trials at the Medical University of Vienna. We will focus on those data elements referenced in the trials that are covered by the EHR4CR list and check, to what extent the corresponding ELGA CDA components are actually documented.

## Data Availability

Our concept repository “EHR4CR Data Inventory” is publicly accessible at https://art-decor.org/ad/#/ehr4cr-/project/overview. Our mappings of the concept repository to ELGA can be found at https://art-decor.org/ad/#/ehr4cr2elga-tf-/project/overview (use case “feasibility checking”), at https://art-decor.org/ad/#/ehr4cr2elga-pr-/project/overview (use case “patient identification and recruitment”), and at https://art-decor.org/ad/#/ehr4cr2elga-te-/project/overview (use case “trial execution”).Our applications for the automated matching of the analysis of the EHR4CR Data Inventory concepts to the EHR system’s CDA templates and for the automatic analysis of the template mappings are available from https://gitlab.com/muv-mim/ehr4cr2ehr.

## References

[1] Prokosch HU, Ganslandt T. Perspectives for medical informatics. Reusing the electronic medical record for clinical research. Methods Inf Med. 2009;48:38–44. 19151882

[2] Rogers JR, Lee J, Zhou Z, Cheung YK, Hripcsak G, Weng C. Contemporary use of real-world data for clinical trial conduct in the United States: a scoping review. J Am Med Inf Assoc. 2021;28:144–54. 10.1093/jamia/ocaa224. 10.1093/jamia/ocaa224 PMC7810452 33164065

[3] Becker L, Ganslandt T, Prokosch HU, Newe A. Applied practice and possible leverage points for information technology support for patient screening in clinical trials: qualitative study. JMIR Med Inf. 2020;8:e15749. 10.2196/15749. 10.2196/15749 PMC7327588 32442156

[4] Kopcke F, Prokosch HU. Employing computers for the recruitment into clinical trials: a comprehensive systematic review. J Med Internet Res. 2014;16. 10.2196/jmir.3446. e161. 10.2196/jmir.3446 PMC4128959 24985568

[5] El Fadly A, Rance B, Lucas N, Mead C, Chatellier G, Lastic PY, et al. Integrating clinical research with the healthcare enterprise: from the RE-USE project to the EHR4CR platform. J Biomed Inform. 2011;44(1):94–102. 10.1016/j.jbi.2011.07.007. 21888989 10.1016/j.jbi.2011.07.007

[6] Kopcke F, Trinczek B, Majeed RW, Schreiweis B, Wenk J, Leusch T, et al. Evaluation of data completeness in the electronic health record for the purpose of patient recruitment into clinical trials: a retrospective analysis of element presence. BMC Med Inf Decis Mak. 2013;13:37. 10.1186/1472-6947-13-37. 10.1186/1472-6947-13-37 PMC3606452 23514203

[7] Ateya MB, Delaney BC, Speedie SM. The value of structured data elements from electronic health records for identifying subjects for primary care clinical trials. BMC Med Inf Decis Mak. 2016;16:1. 10.1186/s12911-016-0239-x. 10.1186/s12911-016-0239-x PMC4709934 26754574

[8] Rasmussen LV, Brandt PS, Jiang G, Kiefer RC, Pacheco JA, Adekkanattu P, et al. Considerations for improving the portability of electronic health record-based phenotype algorithms. AMIA Annual Symp Proc / AMIA Symp AMIA Symp. 2019;2019:755–64. PMC7153055 32308871

[9] De Moor G, Sundgren M, Kalra D, Schmidt A, Dugas M, Claerhout B, et al. Using electronic health records for clinical research: the case of the EHR4CR project. J Biomed Inform. 2015;53:162–73. 10.1016/j.jbi.2014.10.006. 25463966 10.1016/j.jbi.2014.10.006

[10] Califf RM. The patient-centered outcomes research network: a national infrastructure for comparative effectiveness research. N C Med J. 2014;75:204–10. 10.18043/ncm.75.3.204 24830497 10.18043/ncm.75.3.204

[11] Hripcsak G, Duke JD, Shah NH, Reich CG, Huser V, Schuemie MJ, et al. Observational health data sciences and informatics (OHDSI): opportunities for observational researchers. Stud Health Technol Inf. 2015;216:574–8. PMC4815923 26262116

[12] Kohane IS, Churchill SE, Murphy SN. A translational engine at the national scale: informatics for integrating biology and the bedside. J Am Med Inf Assoc. 2012;19:181–5. 10.1136/amiajnl-2011-000492 10.1136/amiajnl-2011-000492 PMC3277623 22081225

[13] Liu H, Chi Y, Butler A, Sun Y, Weng C. A knowledge base of clinical trial eligibility criteria. J Biomed Inform. 2021;117:103771. 10.1016/j.jbi.2021.103771. 33813032 10.1016/j.jbi.2021.103771 PMC8407851

[14] Si Y, Weng C. An OMOP CDM-based relational database of clinical research eligibility criteria. Stud Health Technol Inf. 2017;245:950–4. PMC5893219 29295240

[15] International Organization for Standardization. ISO/TR 20514:2005 Health informatics -- Electronic health record -- Definition, scope and context. In: Standardization IOf; 2005.

[16] World Health Organization. Digital Health in the European Region: the ongoing journey to commitment and transformation. 2023. https://www.who.int/europe/publications/i/item/9789289060226. Accessed 17 Feb 2025.

[17] Bertelsmann Stiftung. SmartHealthSystems - International comparison of digital strategies. 2019. https://www.bertelsmann-stiftung.de/en/publications/publication/did/smarthealthsystems-1. Accessed 18 Feb 2025.

[18] Dolin RH, Alschuler L, Boyer S, Beebe C, Beilen FM, Biron PV, et al. HL7 clinical document architecture, release 2. J Am Med Inf Assoc. 2006;13:30–9. 10.1197/jamia.M1888 PMC1380194 16221939

[19] Torab-Miandoab A, Samad-Soltani T, Jodati A, Rezaei-Hachesu P. Interoperability of heterogeneous health information systems: a systematic literature review. BMC Med Inf Decis Mak. 2023;23:18. 10.1186/s12911-023-02115-5. 10.1186/s12911-023-02115-5 PMC9875417 36694161

[20] Herbek S, Eisl HA, Hurch M, Schator A, Sabutsch S, Rauchegger G, et al. The Electronic Health Record in Austria: a strong network between health care and patients. Eur Surg. 2012;155–63. 10.1007/s10353-012-0092-9

[21] Augustinov G, Duftschmid G. Can the Austrian Nation-Wide EHR system support the recruitment of trial patients? Stud Health Technol Inf. 2019;259:87–90. 30923279

[22] Doods J, Botteri F, Dugas M, Fritz F. A European inventory of common electronic health record data elements for clinical trial feasibility. Trials. 2014;15:18. 10.1186/1745-6215-15-18. 24410735 10.1186/1745-6215-15-18 PMC3895709

[23] Doods J, Lafitte C, Ulliac-Sagnes N, Proeve J, Botteri F, Walls R, et al. A European inventory of data elements for patient recruitment. Stud Health Technol Inf. 2015;210:506–10. 25991199

[24] Bruland P, McGilchrist M, Zapletal E, Acosta D, Proeve J, Askin S, et al. Common data elements for secondary use of electronic health record data for clinical trial execution and serious adverse event reporting. BMC Med Res Methodol. 2016;16:159. 10.1186/s12874-016-0259-3. 10.1186/s12874-016-0259-3 PMC5118882 27875988

[25] Dugas M, Neuhaus P, Meidt A, Doods J, Storck M, Bruland P, et al. Portal of medical data models: information infrastructure for medical research and healthcare. Database: J Biol Databases Curation. 2016;2016. 10.1093/database/bav121. 10.1093/database/bav121 PMC4750548 26868052

[26] Bruland P. Data Inventory for Clinical Trial Execution in the EHR4CR project. 2018. https://medical-data-models.org/29819?lang=en. Accessed 12 Sept 2023.

[27] Doods JBF, Dugas M, Fritz F. A European inventory of common electronic health record data elements for clinical trial feasibility. 2021. https://medical-data-models.org/44179. Accessed 12 Sept 2023. 10.1186/1745-6215-15-18 PMC3895709 24410735

[28] Institut of Medical Informatics WM. EHR4CR data inventory for patient identification and recruitment. 2021. https://medical-data-models.org/44772?lang=en. Accessed 12 Sept 2023.

[29] The ART-DECOR Expert Group. ART-DECOR. 2023. http://art-decor.org. Accessed 12 Sept 2023.

[30] Medical University of Vienna. EHR4CR Data Inventory. 2019. https://art-decor.org/ad/#/ehr4cr-/project/overview. Accessed 25 Jan 2024.

[31] Heitmann KU, Curry J, Nelson L. HL7 Templates Standard: Specification and Use of Reusable Information Constraint Templates, Release 1. 2014.

[32] Reich C, Ostropolets A, Ryan P, Rijnbeek P, Schuemie M, Davydov A, et al. OHDSI standardized vocabularies-a large-scale centralized reference ontology for international data harmonization. J Am Med Inf Assoc. 2024;31:583–90. 10.1093/jamia/ocad247 10.1093/jamia/ocad247 PMC10873827 38175665

[33] Banda JM. OHDSI Ananke - A Tool for Mapping Between OHDSI Concept Identifiers to Unified Medical Language System (UMLS) identifiers. 2020. https://github.com/thepanacealab/OHDSIananke. Accessed 6 Dec 2024.

[34] Katsch F, Duftschmid G. ehr4cr2ehr. 2024. https://gitlab.com/muv-mim/ehr4cr2ehr Accessed 6 Dec 2024.

[35] Medical University of Vienna. EHR4CR2ELGA-trial feasibility. 2022. https://art-decor.org/ad/#/ehr4cr2elga-tf-/project/overview. Accessed 25 Jan 2024.

[36] Medical University of Vienna. EHR4CR2ELGA-patient recruitment. 2022. https://art-decor.org/ad/#/ehr4cr2elga-pr-/project/overview. Accessed 25 Jan 2024.

[37] Medical University of Vienna. EHR4CR2ELGA-trial execution. 2022. https://art-decor.org/ad/#/ehr4cr2elga-te-/project/overview. Accessed 25 Jan 2024.

[38] Heitmann KU, Sabutsch S, Kuttin O, Klostermann A, Leder S, Winkler S, et al. ELGA Repository. 2023. https://art-decor.org/art-decor/decor-project--elgabbr-. Accessed 12 Sept 2023.

[39] Sabutsch S, Heitmann KU, Klostermann A, Kuttin O, Frohner M, Krondraf N et al. ELGA generischer Arztbrief. 2022. https://art-decor.org/art-decor/decor-project--elgagab-. Accessed 12 Sept 2023.

[40] Sabutsch S, Klostermann A, Kuttin O, Sjencic N, Rainer-Sablatnig S, Heitmann KU, et al. AT-CDA-BBR (Allgemeiner ILF). 2023. https://art-decor.org/art-decor/decor-project--at-cda-bbr-. Accessed 12 Sept 2023.

[41] Melzer G, Maiwald T, Prokosch HU, Ganslandt T. Leveraging real-world data for the selection of relevant eligibility criteria for the implementation of electronic recruitment support in clinical trials. Appl Clin Inf. 2021;12:17–26. 10.1055/s-0040-1721010. 10.1055/s-0040-1721010 PMC7806423 33440429

[42] Butler A, Wei W, Yuan C, Kang T, Si Y, Weng C. The Data Gap in the EHR for Clinical Research Eligibility Screening. AMIA Joint Summits on Translational Science proceedings AMIA Joint Summits on Translational Science. 2018;2017:320–9. PMC5961795 29888090

[43] Health Level Seven (HL7). HL7 FHIR Release 5. 2023. https://www.hl7.org/fhir/. Accessed 25 Jan 2024.

[44] Katsch F, Hussein R, Korntheuer R, Duftschmid G. Converting HL7 CDA based nationwide Austrian medication data to OMOP CDM. Stud Health Technol Inf. 2023;302:899–900. 10.3233/shti230300. 10.3233/SHTI230300 37203528

[45] Korntheuer RL, Katsch F, Duftschmid G. Transforming documents of the Austrian nationwide EHR system into the OMOP CDM. Stud Health Technol Inf. 2023;301:54–9. 10.3233/shti230011. 10.3233/SHTI230011 37172152

